# Susceptibility to cigarette smoking among secondary and high school students from a socially disadvantaged rural area in Poland

**DOI:** 10.1186/s12971-016-0092-9

**Published:** 2016-08-15

**Authors:** Kinga Polańska, Piotr Wojtysiak, Leokadia Bąk-Romaniszyn, Dorota Kaleta

**Affiliations:** 1 Department of Tobacco Control, Preventive Medicine Department, Medical University of Lodz, Zeligowskiego 7/8 Street, 90-752 Lodz, Poland; 2 Department of Nutrition in Digestive Tract Diseases, Medical University of Lodz, Lodz, Poland

**Keywords:** Susceptibility to smoking, Adolescents, Students, Socially disadvantaged rural area, Poland

## Abstract

**Background:**

To prevent adolescents from becoming smokers, it is essential to understand factors that cause them to become susceptible to smoking (SS). The aim of this study was to examine association between individual and school characteristics and susceptibility to smoking initiation and experimentation in the youth.

**Methods:**

We collected cross-sectional survey data from students aged 13–19 years attending 21 schools from Piotrkowski district. Of 4050 students, 3552 respondents, including 2508 non-smokers, filled in an anonymous, self-administered questionnaire adapted from the Global Youth Tobacco Survey. The univariate and multivariate logistic regression analyses were applied to the study factors linked to SS among the never and ever smoking youth.

**Results:**

About 22 % of the never smoking and 57 % of the ever smoking students were found to be vulnerable to smoking. The youth who were males (OR = 1.4; *p* = 0.001), who were older (OR = 1.1; *p* = 0.002) and those, whose mothers had medium (OR = 1.8; *p* < 0.001) and lower (OR = 4.1; *p* < 0.001) educational levels were more prone regarding future smoking compared to the females, younger ones and those whose mothers were highly educated. The students who lived in households with no smoking ban (OR = 1.4; *p* = 0.001) and those who had ever tried cigarettes (OR = 3.5; *p* < 0.001) were more susceptible to smoking comparing to those who indicated smoke-free home and who had never smoked. In addition, having smoking friends (OR = 2.3; *p* < 0.001), seeing school personnel smoking on the premises of the school (OR = 1.8; *p* < 0.001) and perceiving smoking girls more attractive than the non-smokers (OR = 3.8; *p* < 0.001) were the correlates of smoking susceptibility. Finally, the separate analysis among the never smokers indicated that no school training on tobacco harm (OR = 1.3; *p* = 0.04) is the additional significant factor for susceptibility to smoking initiation.

**Conclusions:**

SS is prevalent in secondary and high school students in Poland. Personal, social and environmental factors are strongly correlated with SS. When addressing the youth, efforts should be focused on the groups at risk, with a comprehensive approach including multiple factors and involving school personnel, parents and the group leaders in tobacco control activities. Projects aimed at changing social norms around smoking and providing the youth with knowledge and skills to resist smoking are also needed. This may help to implement an effective approach to prevent smoking susceptibility and initiation of tobacco use among the youth.

**Electronic supplementary material:**

The online version of this article (doi:10.1186/s12971-016-0092-9) contains supplementary material, which is available to authorized users.

## Background

Most of the burden of disease attributable to smoking occurs among adults. However, the problem originates in the teenage years when the majority of smokers have their first experience with cigarettes. The existing data indicate that 88 % of adult smokers start smoking before the age of 18 [1]. Simultaneously, those who did not smoke during adolescence are less likely to start smoking later in their life. It is also worth noting that people who started smoking in younger age usually smoke more cigarettes per day, are more strongly addicted to nicotine and are less likely to attempt to quit smoking or if they do so, they have less chance to quit comparing to those who start smoking as adults [2]. Studies have also shown that adolescents who smoke are more prone to additional risky behaviors including the use of alcohol, cannabis and other illicit drugs [3].

Data from The Health Behavior in School-aged Children (HBSC) study, indicate that in Europe, in many countries, every third person aged 13 or younger has been through tobacco initiation [4]. When comparing data from a survey conducted in Poland in 2009/2010 and 2013/2014 a decrease in the percentage of 15-year-old boys who reported their first cigarette at the age of 13 or younger has been observed (from 30 to 26 %) [4, 5]. At the same time, an opposite trend has been noted among girls (an increase from 20 to 22 %). A belief that smoking helps maintain a slim silhouette, fashion, marketing of tobacco companies addressed to females and a positive image of tobacco smoking in the media all constitute factors that significantly contribute to the reduction of age at the moment of tobacco initiation among girls population in Poland [6].

It should be underlined that cigarette smoking is a learned behavior, which passes through various stages namely: preparation, initiation, experimentation, regular and long-term smoking and addiction [7]. Susceptibility to smoking defined as lack of a firm commitment not to smoke is, therefore, useful for predicting which young people may become smokers. The susceptible youth are more likely to experiment with tobacco and to become regular smokers than the non-susceptible youth [8]. According to the existing data considering smoking, susceptibility has been shown to be modifiable through interventions [9]. Taking this into consideration when addressing the youth, efforts should either prevent target groups of non–smokers from becoming susceptible to smoking or prevent susceptible adolescents from progressing to regular smokers.

A growing literature has documented various factors that influence susceptibility to smoking such as: individual characteristics (e.g. age, gender), immediate social environment (e.g. friends and family) and broader social environment (e.g. school) [10–16]. Nevertheless, there is still a need to extend knowledge on this topic. Monitoring the prevalence and identification of smoking susceptible individuals will allow development of preventive measures to be taken to reduce smoking initiation among young people and consequently to reduce smoking prevalence. Evidence of prevalence and factors associated with the youth smoking susceptibility, although crucial for developing and implementing effective tobacco control strategies, is limited in Poland, especially in relation to a rural population.

Thus, the aim of this study was to examine the association between individual and school characteristics and smoking susceptibility in the non-currently smoking youth.

## Methods

### Study design and population

During the period from November 2014 and May 2015 a cross-sectional study was conducted among secondary and high school students from Piotrkowski district (aged 13–19 years), which is a socially disadvantaged rural area in central Poland (Lodzkie voivodeship). In accordance with the state of 31.12.2013, on the premises of the district there were 91,618 residents. More than 90 % of the residents of the district were people who lived in a rural area (villages with fewer than 10, 000 residents). Results of the analysis conducted in 2012 by the United Nations Development Programme, placed Piotrkowski district among 30 districts, of all 314 that exist in Poland, with the lowest indicators of social development. Local Human Development Index covering three indicators: Health Index, Education Index, Welfare Index was 25.97 (including Health Index HI = 26.50), whereas the discussed indicators for Lodzkie voivodeship were, respectively: 39.28 and 31.48 [17]. The above data indicate a low level of socio-economic development of Piotrkowski district.

Before the study commenced, approvals of the Education Management Centre in Piotrkowski district and of directors of educational institutions where the research was to be carried out were obtained. All of the 16 secondary and 5 high schools in Piotrkowski district were invited to participate in the project and all of them agreed to being involved in the study.

In total, 4050 students were invited to participate. Of this group, the filled in questionnaires were returned by 3552 respondents (88 %). The characteristics of the sample is provided in supplementary materials (Additional file 1: Table S1). As the aim of the study was to assess the patterns of smoking susceptibility, current smokers defined as the students who had smoked at least once in the past 30 days were excluded from the analysis. The participants were also excluded due to missing data. Finally, the study population consisted of 2508 students including 1425 never smokers (these who had never tried smoking in their lifetime) and 1083 ever smokers (these who had ever smoked even once one or two puffs in the past).

The study obtained a positive opinion from the Bioethics Committee of Medical University in Lodz [decision number: RNN/730/14/KB]. A written informed consent was obtained from all the participants aged 18 or above or from parents/legal guardians of the under aged respondents.

### Questionnaire and study measures

An anonymous, self-administrated questionnaire filled in by the students during regular classes, consisted of 84 questions, including core questions from the original Global Youth Tobacco Survey (GYTS) questionnaire, and additional country-specific questions (that mostly focused on the legislation and its enforcement in Poland).

The measure of susceptibility to smoking was adopted from Pierce et al. (1996), which consisted of two questions: 1) “If one of your friends offered you a cigarette, would you smoke it?”, 2) “At any time during the next twelve months, do you think you will smoke a cigarette?” [8]. The students who answered definitely not to both questions were coded as non-susceptible and all other were considered as susceptible to smoking.

Independent variables included the following socio-demographic data: gender, age, school grade and parental education (high: over 12 years of education (university degree), medium: 9–12 years of education (secondary school), low: 9 years or less (primary/vocational school)). The question on parents’ smoking was asked separately, and the students were categorized as children of neither smokers vs. children of one or both parents being smokers. The friends’ smoking status was assessed based on the following question “Do any of your closest friends smoke tobacco?” with possible answers: a) non, b) some of them, c) most of them or d) all of them (with b, c and d combined in the current analysis into one category: have friends who smoke). The respondents were also asked about tobacco smoking ban at home as well as in their school, including enforcement of that regulations. Finally, their perception of smoking by friends and participation in a school training on tobacco harm were noted.

### Statistical analysis

The data were entered into Excel data analysis software on a daily basis by field investigators and submitted to a supervisor. Once the data collection process had been completed, 5 % of the records were randomly checked to confirm that they were clearly recorded, complete and consistent across the responses. Data set is provided in Additional file 2. The univariate and multivariate logistic regression analyses with the results being presented as odds ratios (OR) with 95 % confidence intervals (95 % CI) were applied to study factors linked to susceptibility to smoking among the youth. A separate analysis was additionally performed among the never and the ever smokers. In that perspective susceptibility has been used as a predictor for experimentation with cigarette smoking (the ever smokers) and smoking initiation (the never smokers). First, we ran the univariate logistic regression analyses with susceptibility to smoking as a dependent variable and demographic data, experiences as well as restrictions and attitudes towards tobacco as the independent variables. Variables with *p* values equal or less than 0.1 from the univariate analysis were included in the multivariate one. Next, the backward likelihood step-wise methods of logistic regression was performed to confirm the factors associated with susceptibility to smoking. The final model was checked for fitness using the Hosmer-Lemeshow goodness of fit test. A *p* value of less than 0.05 was considered statistically significant. The STATISTICA Windows XP version 10.0 program was used to carry out the statistical analysis.

## Results

Characteristics of the students included in the current analysis are summarized in Table 1 and in the total sample in Additional file 1: Table S1. Boys represented 54 % of our study sample. Most of the respondents (79 %) were attending secondary schools. About 43 % of the youth indicated that they had ever tried smoking in their lifetime. Similar percentage indicated at least one parent smoking, and more that 75 % had friends who smoke.

**Table 1:** Prevalence of susceptibility to smoking among the secondary and high school students from Piotrkowski district

Characteristic	Total		Susceptible to smoking		Non-susceptible to smoking	
	*N* = 2508		*n* = 925		*n* = 1583	
	*n*	%	*n*	%	*n*	%
Gender						
Male	1361	54.3	556	40.8	805	59.1
Female	1147	45.7	369	32.2	778	67.8
Age						
13	675	26.9	210	31.1	465	68.9
14	685	27.3	241	35.2	444	64.8
15	621	24.8	235	37.8	386	62.2
16	144	5.7	68	47.2	76	52.8
17	124	4.9	57	46.0	67	54.0
18	109	4.4	43	39.4	66	60.6
19	150	6.0	71	47.3	79	52.7
School grade						
1st of secondary school	675	26.9	210	31.1	465	68.9
2nd of secondary school	685	27.3	241	35.2	444	64.8
3rd of secondary school	621	24.8	235	37.8	386	62.2
1st of high school	165	6.6	76	46.1	89	53.9
2nd of high school	138	5.5	59	43.7	79	58.5
3rd of high school	224	8.9	104	46.4	120	53.6
Father’s education						
Low	1484	59.2	614	41.4	870	58.6
Medium	599	23.9	187	312	412	68.8
High	425	16.9	124	29.2	301	70.8
Mother’s education						
Low	1107	44.1	539	48.7	568	51.3
Medium	688	27.4	256	37.2	432	62.8
High	713	28.4	130	18.2	583	81.8
Smoking experience						
Ever smoker	1083	43.2	616	56.9	467	43.1
Never smoker	1425	56.8	309	21.7	1116	78.3
Parental smoking						
None	1447	57.7	475	32.8	972	67.2
One or both parents	1061	42.3	450	42.4	611	57.6
Friends’ smoking status						
Don’t have friends who smoke	604	24.1	112	18.5	492	81.5
Some friends smoke	1368	54.8	531	38.8	837	61.2
Most of the friends or all of them smoke	536	21.4	282	52.6	254	47.4
Seen people using tobacco when watched TV, videos, or movies						
Yes	2184	87.1	807	36.9	1377	63.1
No	324	21.9	118	36.4	206	63.6
Smoking ban at home						
Yes	1090	43.5	313	28.7	777	71.3
No	1418	56.5	612	43.2	806	56.8
Smoke free school						
Yes	1613	64.3	586	36.3	1027	63.7
No	895	35.7	339	37.9	556	62.1
Ever seen friend smoking on the school premises						
Yes	2262	90.2	856	37.8	1406	62.2
No	246	9.8	69	28.1	177	71.9
Ever seen school personnel smoking on the school premises						
Yes	1908	76.1	768	40.3	1140	59.7
No	600	23.9	157	26.2	443	73.8
School training on tobacco harm						
Yes	1249	49.8	392	31.4	857	68.6
No	1259	50.2	533	42.3	726	57.7
Boys who smoke are more or less attractive						
Less attractive or no difference	2420	96.5	875	36.2	1545	63.8
More attractive	88	3.5	50	56.8	38	43.2
Girls who smoke are more or less attractive						
Less attractive or no difference	2389	95.3	836	35.0	1553	65.0
More attractive	119	4.7	89	74.8	30	25.1

In our study population 37 % of the non-currently smoking students were found to be susceptible to smoking including 41 % among boys and 32 % among girls. Higher percentage of the smoking susceptible youth was observed among older students (47 % among 19-year-old people comparing to 31 % among 13-year-old ones). The students whose parents had the highest educational level were less susceptible to smoking than those whose parents were in the lowest educational level category (for mothers 18 % vs. 49 %; for fathers 29 % vs. 41 %). A higher percentage of the young people who were susceptible to smoking was observed among the ever than the never smokers (57 % vs. 22 %).

In the group of the youth, whose parents and friends smoked tobacco, those susceptible to smoking were found more frequently than in the group with non-smoking relatives. A similar pattern was noted for the smoking ban at home (in the group of the students who indicated existence of such a regulation, the percentage of susceptible to smoking young people was lower than that in the group who declared that they did not have such restrictions at home: 29 % vs. 43 %). Such differences were not observed when considering smoke free school regulations. Among the students who had seen friend or school personnel smoking on the school premises, the percentage of teenagers susceptible to smoking was higher (38 and 40 % respectively) than among those who had never seen people smoking in school or on the premises of the school (28 and 26 %). Similarly, the youth who thought that those who smoke are more attractive, seemed to be susceptible to smoking more frequently than those who did not share that opinion.

The univariate analysis indicated that male gender, older age and having parents with a lower educational level lead to a higher risk of susceptibility to smoking (gender: OR = 1.5, *p* < 0.001; age: OR = 1.2; *p* < 0.001; mother education: OR = 4.3; *p* < 0.001; father education: OR = 1.7; *p* < 0.001) (Table 2). The youth were also more likely to be susceptible to smoking if they had ever tried cigarettes, and had at least one parent or a friend who smoked (OR = 4.8; OR = 1.5; OR = 3.3; *p* < 0.001, respectively). The students who had ever seen school personnel smoking on the school premises (OR = 1.9; *p* < 0.001), those who indicated no classes on harmful effects of smoking (OR = 1.6; p < 0.001) and those having perceptions that smoking teenagers are more attractive than the non-smokers (OR = 2.3 and OR = 5.5; *p* < 0.001) were also more likely to be susceptible to smoking. The student’s susceptibility to smoking was not significantly associated with smoke free school regulations, seeing friends smoking on the school premises as well as people using tobacco in watched TV, videos or movies (*p* > 0.05).

**Table 2:** Factors associated with susceptibility to smoking among the secondary and high school students from Piotrkowski district

Characteristic	Crude			Adjusted		
	OR	95 % CI	*p*-value	OR	95 % CI	*p*-value
Gender						
Male	1.46	1.24–1.72	0.0001	1.39	1.15–1.67	0.001
Female	1.00	Ref.		1.00	Ref.	
Age in years (continuous variable)	1.23	1.07–1.18	0.0001	1.09	1.03–1.15	0.002
Father’s education						
Low	1.71	1.36–2.16	0.0001			
Medium	1.10	0.84–1.44	0.5			
High	1.00	Ref.				
Mother’s education						
Low	4.26	3.40–5.32	0.0001	4.10	3.20–5.25	0.0001
Medium	2.66	2.08–3.10	0.0001	1.84	1.41–2.41	0.0001
High	1.00	Ref.		1.00	Ref.	
Smoking experience						
Ever smoker	4.76	4.00–5.67	0.0001	3.51	2.89–4.26	0.0001
Never smoker	1.00	Ref.		1.00	Ref.	
Parental smoking						
None	1.00	Ref.				
One or both parents	1.51	1.28–1.78	0.0001			
Friends’ smoking status						
Don’t have friends who smoke	1.00	Ref.		1.00	Ref.	
Have friends who smoke	3.27	2.61–4.10	0.0001	2.30	1.78–2.96	0.0001
Seen people using tobacco when watched TV, videos, or movies						
Yes	1.02	0.80–1.30	0.8			
No	1.00	Ref.				
Smoking ban at home						
Yes	1.00	Ref.		1.00	Ref.	
No	1.88	1.59–2.23	0.0001	1.38	1.14–1.67	0.001
Smoke free school						
Yes	1.00	Ref.				
No	1.07	0.90–1.27	0.4			
Ever seen friend smoking on the school premises						
Yes	1.13	0.96–1.34	0.1			
No	1.00	Ref.				
Ever seen school personnel smoking on the school premises						
Yes	1.90	1.55–2.33	0.0001	1.81	1.42–2.31	0.0001
No	1.00	Ref.		1.00	Ref.	
School training on tobacco harm						
Yes	1.00	Ref.				
No	1.61	1.36–1.89	0.0001			
Boys who smoke are more or less attractive						
Less attractive or no difference	1.00	Ref.				
More attractive	2.32	1.51–3.57	0.0001			
Girls who smoke are more or less attractive						
Less attractive or no difference	1.00	Ref.		1.00	Ref.	
More attractive	5.51	3.61–8.41	0.0001	3.83	2.40–6.13	0.0001

The further analysis using the multiple regression also provided consistent results (Table 2). The youth who were males (OR = 1.4; *p* = 0.001) and the older ones (OR = 1.1; *p* = 0.002) were more likely to be susceptible to smoking as compared to the females and younger ones. The risk of smoking susceptibility was also higher for those whose mothers had medium (OR = 1.8; *p* < 0.001) and lower (OR = 4.1; *p* < 0.001) educational levels than in the group whose mothers were highly educated. The students who declared that there were no smoking bans at their home (OR = 1.4; *p* = 0.001) and those who had ever tried cigarettes (OR = 3.5; *p* < 0.001) were susceptible to smoking comparing to those who indicated smoke-free home and who had never smoked. Finally, having smoking friends (OR = 2.3; *p* < 0.001), seeing school personnel smoking on the school premises (OR = 1.8; *p* < 0.001) and having perception that smoking girls are more attractive than the non-smokers (OR = 3.8; *p* < 0.001) increased the risk of smoking susceptibility.

Finally, a stratify analysis has been performed based on ever smoking and never smoking categories (Tables 3 and 4 and Additional file 1: Table S2). Among the ever smokers 57 % and among the never smokers 22 % were susceptible to smoking. We found that students who were ever smokers and were boys (OR = 1.7; *p* < 0.001), had mothers with a lower educational level (OR = 6.0; *p* < 0.001), had smoking parents (OR = 1.5; *p* < 0.001) and friends who smoked (OR = 2.9; *p* < 0.001) and these who shared the opinion that girls who smoke were more attractive (OR = 7.2; *p* < 0.001) were more likely to be susceptible to smoking experimentation. Never smokers whose mothers had a lower educational level (OR = 2.8; *p* < 0.001), had smoking friends (OR = 2.5; *p* < 0.001), declared no smoking ban at home (OR = 1.5; *p* = 0.002) as well as no school training on tobacco harm (OR = 1.3; *p* = 0.04) and who shared the opinion that boys who smoke were more attractive (OR = 2.5; *p* < 0.001), were more likely to be susceptible to smoking initiation.

**Table 3:** Factors associated with susceptibility to smoking among the secondary and high school students from Piotrkowski district - analysis for the never smokers

Characteristic	Crude			Adjusted		
	OR	95 % CI	*P*-value	OR	95 % CI	*P*-value
Gender						
Male	1.28	0.99–1.64	0.06			
Female	1.00	Ref.				
Age in years (continuous variable)	1.09	1.01–1.18	0.03			
Father’s education						
Low	1.88	1.29–2.73	0.001			
Medium	1.55	1.03–2.33	0.04			
High	1.00	Ref.				
Mother’s education						
Low	2.93	2.13–4.03	<0.001	2.79	1.99–3.90	<0.001
Medium	1.93	1.31–2.82	<0.001	1.67	1.12–2.48	0.01
High	1.00	Ref.		1.00	Ref.	
Parental smoking						
None	1.00	Ref.				
One or both parents	1.47	1.14–1.89	0.003			
Friends’ smoking status						
Don’t have friends who smoke	1.00	Ref.		1.00	Ref.	
Have friends who smoke	2.92	2.10–4.03	<0.001	2.53	1.81–3.55	<0.001
Seen any people using tobacco when watched TV, videos, or movies						
Yes	1.27	0.87–1.86	0.21			
No	1.00	Ref.				
Smoking ban at home						
Yes	1.00	Ref.		1.00	Ref.	
No	2.00	1.54–2.60	<0.001	1.54	1.17–2.04	0.002
Smoke free school						
Yes	1.00	Ref.				
No	1.16	0.88–1.52	0.29			
Ever seen friend smoking on the school premises						
Yes	2.39	1.40–4.09	0.001			
No	1.00	Ref.				
Ever seen school personnel smoking on the school premises						
Yes	2.05	1.51–2.77	<0.001			
No	1.00	Ref.				
School training on tobacco harm						
Yes	1.00	Ref.		1.00	Ref.	
No	1.47	1.14–1.90	0.003	1.34	1.02–1.77	0.04
Boys who smoke are more or less attractive						
Less attractive or no difference	1.00	Ref.		1.00	Ref.	
More attractive	4.00	2.10–7.59	<0.001	2.52	1.82–3.48	<0.001
Girls who smoke are more or less attractive						
Less attractive or no difference	1.00	Ref.				
More attractive	1.45	0.56–3.78	0.44

**Table 4:** Factors associated with susceptibility to smoking among the secondary and high school students from Piotrkowski district - analysis for the ever smokers

Characteristic	Crude			Adjusted		
	OR	95 % CI	*P*-value	OR	95 % CI	*P*-value
Gender						
Male	1.49	1.17–1.90	0.001	1.73	1.32–2.29	<0.001
Female	1.00	Ref.		1.00	Ref.	
Age in years (continuous variable)	1.05	0.98–1.12	0.18			
Father’s education						
Low	1.16	0.82–1.65	0.40			
Medium	0.96	0.62–1.47	0.84			
High	1.00	Ref.				
Mother’s education						
Low	6.03	4.25–8.54	<0.001	5.95	4.11–8.61	<0.001
Medium	2.22	1.57–3.15	<0.001	2.15	1.49–3.09	<0.001
High	1.00	Ref.		1.00	Ref.	
Parental smoking						
None	1.00	Ref.		1.00	Ref.	
One or both parents	1.55	1.22–1.98	<0.001	1.47	1.11–1.95	<0.001
Friends’ smoking status						
Don’t have friends who smoke	1.00	Ref.		1.00	Ref.	
Have friends who smoke	2.00	1.40–2.84	<0.001	2.85	1.90–4.27	<0.001
Seen any people using tobacco when watched TV, videos, or movies						
Yes	1.11	0.94–1.52	0.18			
No	1.00	Ref.				
Smoking ban at home						
Yes	1.00	Ref.				
No	1.41	1.10–1.81	0.007			
Smoke free school						
Yes	1.00	Ref.				
No	1.14	0.83–1.79	0.33			
Ever seen friend smoking on the school premises						
Yes	1.22	0.81–1.64	0.35			
No	1.00	Ref.				
Ever seen school personnel smoking on the school premises						
Yes	1.09	0.87–1.24	0.22			
No	1.00	Ref.				
School training on tobacco harm						
Yes	1.00	Ref.				
No	0.95	0.74–1.22	0.71			
Boys who smoke are more or less attractive						
Less attractive or no difference	1.00	Ref.				
More attractive	1.21	0.67–2.17	0.53			
Girls who smoke are more or less attractive						
Less attractive or no difference	1.00	Ref.		1.00	Ref.	
More attractive	4.69	2.67–8.25	<0.001	7.17	2.81–9.52	<0.001

## Discussion

Susceptibility to cigarette smoking is prevalent among secondary and high school students in Poland. Identification of factors that predict the probability of smoking in the future is an important public health challenge. The current study contributes to the growing body of literature about the role of various factors that influence susceptibility to smoking among young people from a socially disadvantaged rural area. In particular, males, those who were older, who had ever tried cigarettes and whose mothers had a lower educational level were more prone to future smoking. In addition, living in households with no smoking ban, having smoking friends, seeing school personnel who smoke and perceiving smoking girls as more attractive were the important correlates of smoking susceptibility. The separate analysis among the never smokers indicated that no school training on tobacco harm is an additional significant factor for susceptibility to smoking initiation.

The prevalence of susceptibility to smoking observed in our assessment (22 % among the never smokers and 57 % among the ever smokers) is higher than the one noted in the studies conducted in China (6–7 %), Thailand (9 %), Taiwan (11 %), Pakistan (12 %), Malaysia (16 %), and it is closer to the results obtained in the USA (21 %) and Canada (about 30 %) [10–12, 18–20]. Cross-sectional data for 168 countries obtained from GYTS have indicated that approximately 1 in 8 never-smoking youth worldwide was found to be susceptible to smoking with wide differences between the WHO regions (with the highest proportion of the youth susceptible to smoking observed in Europe and the Americas) [13]. An additional analysis of GYTS data from 25 European countries has confirmed high susceptibility to smoking among the youth (including almost every fourth person susceptible to smoking in Poland, which is in agreement with the results obtained in our study) [21]. The differences between the studies concerning the proportion of those susceptible to smoking can result from many reasons, including: social and cultural norms, tobacco industry influences and legislation as well as prevention activities. In addition, the age of population included, definition of non-smoking (never/ever smokers) and the region in which the study was conducted (urban/ruler) can be responsible for inconsistencies between the studies.

Despite the well-documented adverse health effects of tobacco use, about 23 % of young males and 12 % of young females (aged 15–19) in Poland are current smokers (including 16 % of daily smoking males and 8 % of daily smoking females) [22]. In this age group, among non-daily and non-occasional smokers, about 30 % smoked at least once in their lifetime. In addition, as it was mentioned previously, more than 20 % of teenagers reported their first cigarette at the age of 13 or younger [4]. These results, together with our assessments, confirm the need for an intervention either to prevent target groups of non–smokers from becoming susceptible to smoking or to prevent susceptible adolescents from progressing to regular smokers, which, in the end, can lead to a reduction of smoking prevalence. In that perspective, the current study indicating the group at risk could be crucial for developing and implementing effective tobacco control strategies.

Our results showed that gender (males) and older age were positively associated with susceptibility to smoking, which is mostly observed among ever smokers. Existing research has reported less consistent associations between susceptibility to smoking and gender. In some studies, similarly to our results, males have been more susceptible to smoking [11, 12, 23, 24], whereas in other studies, opposite outcomes have been observed [10, 18, 25–27]. This phenomenon may be due to socio-cultural differences between the countries and it can also indicate to what extent young people are influenced and susceptible to tobacco marketing [11]. Most of the existing research indicates that older age is the risk factor for smoking susceptibility, experimentation with smoking and smoking habit [16, 28–31], and our results are in line with those observations.

In general, there is a strong association between parents’ and friends’ smoking status and smoking initiation among adolescents [10–12, 32, 33]. We found friends’ smoking status to be a stronger predictor of susceptibility to smoking among the never smokers than the parental smoking status. It is proven that people tend to choose their friends based on shared characteristics, including tobacco smoking [34]. However, having close friends who smoke does not need to mean that they cause the person to smoke. On the other hand, strong commitment not to smoke if offered a cigarette by a friend is crucial as a protective factor for not starting smoking [16]. In our assessment perceiving smoking boys as more attractive ones than those non-smoking constitutes an important correlate of susceptibility to smoking initiation (in the analysis among the never smokers). What is interesting, among the youth who have already experimented with smoking the opinion that smoking girls are more attractive than the non-smoking ones is significantly related to smoking susceptibility. This suggests that peer context in which the youth find themselves plays an important role in smoking susceptibility and warrants further attention in anti-smoking activities dedicated to young people. Prevention should mostly focus on creating non-smoking fashion and enforcing existing legislation. Our results in this context are consistent with Hock et al. (2013), Leatherdale et al. (2015) and Chen et al. (2013) whose studies report that school environment can cause a school to be a significant risk place for smoking initiation [11, 35, 36]. It also needs to be pointed out that in our assessment the never smokers who indicated no school training on tobacco harm and those with no home smoking restrictions were more likely to initiate smoking. This is in line with previous studies that have found tobacco control measures (including smoke-free home) and health education to be preventive of smoking susceptibility [10–12].

We found that the ever-smokers had more than three times higher risk of susceptibility to smoking as compared to those who had never smoked. These findings are consistent with other results and might be explained by the observation that previous habits were kept and would influence future behavior [10, 11, 29]. It has been also proven by Buller et al. (2003) who have indicated that past smokers had more friends who smoked, had a positive disposition towards smoking as well as were less concerned about the negative physical and social consequences and these would contribute to their being susceptible to smoking [37].

### Study limitations and strengths

This study is the most recent report on the association of various risk factors with susceptibility to smoking among Polish youth. The data used in the current analysis are based on a large number of respondents from the entire area of Piotrkowski district, assuring generalizability of the results for rural areas; however, its applicability to urban areas may be limited. The study protocol and questionnaire are based on valid tools and GYTS standards developed by experts in the field, which enables comparison between countries and trends assessments [1]. The study has some limitations that need to be pointed. Firstly, all estimates in our assessment were based on self-reports, which might be affected by reporting bias. Secondly, due to the cross-sectional nature of the study, claims of causation cannot be made about the observed relationships between susceptibility to smoking and the studied variables. Moreover, due to a cross-sectional scheme with no repeated observations, it is not possible to capture changes in the intensity of influence of selected factors on smoking susceptibility and status among the teenagers over time. However, from a public health perspective, our data may be a sufficient reason to take preventive actions at local and national levels. Finally, our analysis did not control for other substances use such as alcohol or illicit drugs, which are also indicated to be associated with smoking. Despite the mentioned limitations, the current study provides a valuable insight into the prevalence and factors associated with susceptibility to smoking in Poland. Taking into account that Poland is one of the European countries with the highest smoking prevalence, the study findings are crucial for prevention strategies to be taken among the youth. The study is also in line with the Programme of Reduction of Health Consequences of Tobacco Smoking in Poland for the years 2014–2018, one of the aims of which is to increase the percentage of the youth who have never tried smoking.

## Conclusion

The study indicated a high percentage of young people who are susceptible to smoking. Polish teenagers who are males, attend high school, those whose mothers have a lower educational level and those with no smoking ban at home are more likely to be susceptible to smoking. The higher risk of smoking susceptibility was also observed for those who had ever tried cigarettes, seen school personnel smoking on the school premises, had smoking friends and indicated that smoking girls were more attractive than the non-smoking ones. The separate analysis among the never smokers indicated that no school training on tobacco harm is the additional significant factor for susceptibility to smoking initiation.

Preventive efforts in order to make the programs gender and culture sensitive need to focus on various social and behavioral aspects. Schools are the major public institutions in Piotrkowski district. What schools and school staff define as important to the health and well-being of the students reflects out into the communities they serve. Schools that actively promote tobacco-free living make a strong statement that tobacco use is not acceptable. Importance of health education in schools, as an intervention against tobacco smoking, has been found to be an important preventive factor for tobacco initiation, experimentation and smoking. School-based interventions need to be improved further to avail their maximum benefit as a preventive factor. Apart from school-based tobacco programs, there is also a need for combined efforts at all levels. In addition to the enforcement of the existing legislation, prevention should also focus on increasing awareness of law and health consequences of smoking (pointing these which are related to youth population), decreasing social acceptance for smoking and creating a non-smoking fashion. The youth’s attitudes towards smoking and their decisions to stay smoke-free are shaped by many factors in their environment [13, 38]. A comprehensive approach, based on building and supporting protective factors in the youth will reduce susceptibility to smoking as well as other related unhealthy behaviors.

## Additional files

Additional file 1: Table S1. Baseline characteristics of the sample. **Table S2.** Prevalence of susceptibility to smoking among the secondary and high school students from Piotrkowski district. (DOCX 23 kb) Additional file 2: Data set. (XLSX 1501 kb)
