# Supplementary material for: Susceptibility to cigarette smoking among secondary and high school students from a socially disadvantaged rural area in Poland
Source: Tob Induc Dis. 2016 Aug 15;14:28. doi: 10.1186/s12971-016-0092-9 (PMC4986373; doi:10.1186/s12971-016-0092-9)
Supplement: Additional file 1: Table S1. — Baseline characteristics of the sample. Table S2. Prevalence of susceptibility to smoking among the secondary and high school students from Piotrkowski district. (DOCX 23 kb) [file 12971_2016_92_MOESM1_ESM.docx]

Table S1. Baseline characteristics of the sample

|  | Never smokers  n =1425 | | Ever  smokers  n=1083 | | Current smokers  n=1044 | | Total  n=3552 | | Total with missing data  n=4050 | |
| --- | --- | --- | --- | --- | --- | --- | --- | --- | --- | --- |
|  | n | % | n | % | n | % | n | % | N | % |
| Gender | | | | | | | | | | |
| Male | 735 | 51.6 | 626 | 57.8 | 643 | 61.6 | 2004 | 56.4 | 2264 | 55.9 |
| Female | 690 | 48.4 | 457 | 42.2 | 401 | 38.4 | 1548 | 43.6 | 1786 | 44.1 |
| Age | | | | | | | | | | |
| 13 | 447 | 31.4 | 228 | 21.1 | 122 | 11.7 | 797 | 22.4 | 903 | 22.3 |
| 14 | 414 | 29.0 | 271 | 25.0 | 240 | 23.0 | 925 | 26.0 | 1069 | 26.4 |
| 15 | 313 | 22.0 | 308 | 28.4 | 302 | 28.9 | 923 | 26.0 | 1069 | 26.4 |
| 16 | 73 | 5.1 | 71 | 6.6 | 104 | 10.0 | 248 | 7.0 | 275 | 6.8 |
| 17 | 61 | 4.3 | 63 | 5.8 | 103 | 9.9 | 227 | 6.4 | 251 | 6.2 |
| 18 | 57 | 4.0 | 52 | 4.8 | 65 | 6.2 | 174 | 4.9 | 190 | 4.7 |
| 19 | 60 | 4.2 | 90 | 8.3 | 108 | 10.3 | 258 | 7.3 | 292 | 7.2 |
| School grade | | | | | | | | | | |
| 1^st^ of secondary school | 447 | 31.4 | 228 | 21.1 | 122 | 11.7 | 797 | 22.4 | 903 | 22.3 |
| 2^nd^ of secondary school | 414 | 29.0 | 271 | 25.0 | 240 | 23.0 | 925 | 26.0 | 1069 | 26.4 |
| 3^rd^ of secondary school | 313 | 22.0 | 308 | 28.4 | 302 | 28.9 | 923 | 26.0 | 1069 | 26.4 |
| 1^st^ of high school | 84 | 5.9 | 81 | 7.5 | 110 | 10.5 | 275 | 7.7 | 305 | 7.5 |
| 2^nd^ of high school | 68 | 4.8 | 70 | 6.5 | 105 | 10.1 | 243 | 6.8 | 268 | 6.6 |
| 3^rd^ of high school | 99 | 6.9 | 125 | 11.5 | 165 | 15.8 | 389 | 11.0 | 436 | 10.8 |
| Father’s education | | | | | | | | | | |
| Low | 742 | 52.1 | 742 | 68.5 | 699 | 66.9 | 2183 | 61.5 |  |  |
| Medium | 412 | 28.9 | 187 | 17.3 | 187 | 17.9 | 786 | 22.1 |  |  |
| High | 271 | 19.0 | 154 | 14.2 | 158 | 15.1 | 583 | 16.4 |  |  |
| Mother’s education | | | | | | | | | | |
| Low | 619 | 43.4 | 488 | 45.1 | 634 | 60.7 | 1741 | 49.0 |  |  |
| Medium | 313 | 22.0 | 375 | 34.6 | 306 | 29.3 | 994 | 28.0 |  |  |
| High | 493 | 34.6 | 220 | 20.3 | 104 | 10.0 | 817 | 23.0 |  |  |
| Parental smoking | | | | | | | | | | |
| None | 843 | 59.2 | 604 | 55.8 | 400 | 38.3 | 1847 | 52.0 |  |  |
| One or both parents | 582 | 40.8 | 479 | 44.2 | 644 | 61.7 | 1705 | 48.0 |  |  |
| Friends’ smoking status | | | | | | | | | | |
| Don’t have friends who smoke | 459 | 32.2 | 145 | 13.4 | 66 | 6.3 | 670 | 18.9 |  |  |
| Some friends smoke | 785 | 55.1 | 583 | 53.8 | 396 | 37.9 | 1764 | 49.7 |  |  |
| Most of the friends or all of them smoke | 181 | 12.7 | 355 | 32.8 | 582 | 55.8 | 1118 | 31.5 |  |  |
| Seen any people using tobacco when watched TV. videos. or movies | | | | | | | | | | |
| Yes | 1218 | 85.5 | 966 | 89.2 | 910 | 87.2 | 3094 | 87.1 |  |  |
| No | 207 | 14.5 | 117 | 10.8 | 134 | 12.8 | 458 | 12.9 |  |  |
| Smoking ban at home | | | | | | | | | | |
| Yes | 696 | 48.8 | 394 | 36.4 | 314 | 30.1 | 1404 | 39.5 |  |  |
| No | 729 | 51.2 | 689 | 63.6 | 730 | 69.9 | 2148 | 60.5 |  |  |
| Smoke free school | | | | | | | | | | |
| Yes | 1003 | 70.4 | 610 | 56.3 | 602 | 57.7 | 2215 | 62.4 |  |  |
| No | 422 | 29.6 | 473 | 43.7 | 442 | 42.3 | 1337 | 37.6 |  |  |
| Ever seen friend smoking on the school premises | | | | | | | | | | |
| Yes | 1280 | 89.8 | 982 | 90.7 | 963 | 92.2 | 3225 | 90.8 |  |  |
| No | 145 | 10.2 | 101 | 9.3 | 81 | 7.7 | 327 | 9.2 |  |  |
| Ever seen school personnel smoking on the school premises | | | | | | | | | | |
| Yes | 978 | 68.6 | 930 | 85.9 | 935 | 89.6 | 283 | 80.0 |  |  |
| No | 447 | 31.4 | 153 | 14.1 | 109 | 10.4 | 709 | 20.0 |  |  |
| School training on tobacco harm | | | | | | | | | | |
| Yes | 848 | 59.5 | 401 | 37.0 | 328 | 31.4 | 1577 | 44.4 |  |  |
| No | 577 | 40.5 | 682 | 63.0 | 716 | 68.6 | 1975 | 55.6 |  |  |
| Boys who smoke are more or less attractive | | | | | | | | | | |
| Less attractive or no difference | 1386 | 97.3 | 1034 | 95.5 | 963 | 92.2 | 3383 | 95.2 |  |  |
| More attractive | 39 | 2.7 | 49 | 4.5 | 81 | 7.8 | 169 | 4.8 |  |  |
| Girls who smoke are more or less attractive | | | | | | | | | | |
| Less attractive or no difference | 1404 | 98.5 | 985 | 91.0 | 916 | 87.7 | 3305 | 93.0 |  |  |
| More attractive | 21 | 1.5 | 98 | 9.0 | 128 | 12.3 | 247 | 7.0 |  |  |

Table S2. Prevalence of susceptibility to smoking among the secondary and high school students from Piotrkowski district

| Characteristic | | Never smoker  N=1425 | | | | | | | | Ever smoker  N=1083 | | | | | | | |
| --- | --- | --- | --- | --- | --- | --- | --- | --- | --- | --- | --- | --- | --- | --- | --- | --- | --- |
|  |  | Susceptible to smoking n= 309 | | | | Not Susceptible to smoking n=1116 | | | | Susceptible to smoking n=616 | | | | Not Susceptible to smoking  n=467 | | | |
|  |  | n | | % | | n | | % | | n | | % | | n | | % | |
| Gender | | | | | | | | | | | | | | | | | |
| Male | | 174 | | 23.7 | | 561 | | 76.3 | | 382 | | 61.0 | | 244 | | 39.0 | |
| Female | | 135 | | 19.6 | | 555 | | 80.4 | | 234 | | 51.2 | | 223 | | 48.8 | |
| Age | | | | | | | | | | | | | | | | | |
| 13 | | 88 | | 19.7 | | 359 | | 80.3 | | 122 | | 53,5 | | 106 | | 46.5 | |
| 14 | | 76 | | 18.4 | | 338 | | 81.6 | | 165 | | 60.9 | | 106 | | 39.1 | |
| 15 | | 81 | | 25.9 | | 232 | | 74.1 | | 154 | | 50.0 | | 154 | | 50.0 | |
| 16 | | 19 | | 26.0 | | 54 | | 74.0 | | 49 | | 69.0 | | 22 | | 31.0 | |
| 17 | | 18 | | 29.5 | | 43 | | 70.5 | | 39 | | 61.9 | | 24 | | 38.1 | |
| 18 | | 8 | | 14.0 | | 49 | | 86.0 | | 35 | | 67.3 | | 17 | | 32.7 | |
| 19 | | 19 | | 31.7 | | 41 | | 68.3 | | 52 | | 57.8 | | 38 | | 42.2 | |
| School grade | | | | | | | | | | | | | | | | | |
| 1^st^ of secondary school | | 88 | | 19.7 | | 359 | | 80.3 | | 122 | | 53.5 | | 106 | | 46.5 | |
| 2^nd^ of secondary school | | 76 | | 18.4 | | 338 | | 81.6 | | 165 | | 60.9 | | 106 | | 39.1 | |
| 3^rd^ of secondary school | | 81 | | 25.9 | | 232 | | 74.1 | | 154 | | 50.0 | | 154 | | 50.0 | |
| 1^st^ of high school | | 20 | | 23.8 | | 64 | | 76.2 | | 56 | | 69.1 | | 25 | | 30.9 | |
| 2^nd^ of high school | | 19 | | 27.9 | | 49 | | 72.1 | | 40 | | 57.1 | | 30 | | 42.9 | |
| 3^rd^ of high school | | 25 | | 25.3 | | 74 | | 74.7 | | 79 | | 63.2 | | 46 | | 36.8 | |
| Father’s education | | | | | | | | | | | | | | | | | |
| Low | | 182 | | 24.5 | | 560 | | 75.5 | | 432 | | 58.2 | | 310 | | 41.8 | |
| Medium | | 87 | | 21.1 | | 325 | | 78.9 | | 100 | | 53.5 | | 87 | | 46.5 | |
| High | | 40 | | 14.8 | | 231 | | 85.2 | | 84 | | 54.5 | | 70 | | 45.5 | |
| Mother’s education | | | | | | | | | | | | | | | | | |
| Low | | 181 | | 29.4 | | 438 | | 70.8 | | 358 | | 73.4 | | 130 | | 26.6 | |
| Medium | | 67 | | 21.4 | | 246 | | 78.6 | | 189 | | 50.4 | | 186 | | 49.6 | |
| High | | 61 | | 12.4 | | 432 | | 87.6 | | 69 | | 31.4 | | 151 | | 68.6 | |
| Parental smoking | | | | | | | | | | | | | | | | | |
| None | | 160 | | 19.0 | | 683 | | 81.0 | | 315 | | 52.2 | | 289 | | 47.8 | |
| One or both parents | | 149 | | 25.6 | | 433 | | 74.4 | | 301 | | 62.8 | | 178 | | 37.2 | |
| Friends’ smoking status | | | | | | | | | | | | | | | | | |
| Don’t have friends who smoke | | 51 | | 11.1 | | 408 | | 88.9 | | 61 | | 42.1 | | 84 | | 57.9 | |
| Some friends smoke | | 185 | | 23.6 | | 600 | | 76.4 | | 346 | | 59.3 | | 237 | | 40.7 | |
| Most of the friends or all of them smoke | | 73 | | 40.3 | | 108 | | 59.7 | | 209 | | 58.9 | | 146 | | 41.1 | |
| Seen people using tobacco when watched TV, videos, or movies | | | | | | | | | | | | | | | | |  |
| Yes | 271 | | 22.2 | | 947 | | 77.8 | | 536 | | 55.5 | | 430 | | 44.5 | |  |
| No | 38 | | 18.4 | | 169 | | 81.6 | | 80 | | 68.4 | | 37 | | 31.6 | |  |
| Smoking ban at home | | | | | | | | | | | | | | | | |  |
| Yes | 110 | | 15.8 | | 586 | | 84.2 | | 203 | | 51.5 | | 191 | | 48.5 | |  |
| No | 199 | | 27.3 | | 530 | | 72.7 | | 413 | | 59.9 | | 276 | | 40.1 | |  |
| Smoke free school | | | | | | | | | | | | | | | | |  |
| Yes | 210 | | 20.9 | | 793 | | 79.1 | | 376 | | 61.6 | | 234 | | 38.4 | |  |
| No | 99 | | 23.5 | | 323 | | 76.5 | | 240 | | 50.7 | | 233 | | 49.3 | |  |
| Ever seen friend smoking on the school premises | | | | | | | | | | | | | | | | |  |
| Yes | 293 | | 22.9 | | 987 | | 77.1 | | 563 | | 57.3 | | 419 | | 42.7 | |  |
| No | 16 | | 11.0 | | 129 | | 89.0 | | 53 | | 52.5 | | 48 | | 47.5 | |  |
| Ever seen school personnel smoking on the school premises | | | | | | | | | | | | | | | | |  |
| Yes | 246 | | 25.2 | | 732 | | 74.8 | | 552 | | 56.1 | | 408 | | 43.9 | |  |
| No | 63 | | 14.1 | | 384 | | 85.9 | | 94 | | 61.4 | | 59 | | 38.6 | |  |
| School training on tobacco harm | | | | | | | | | | | | | | | | |  |
| Yes | 161 | | 19.0 | | 687 | | 81.0 | | 231 | | 57.6 | | 170 | | 42.4 | |  |
| No | 148 | | 25.6 | | 429 | | 74.5 | | 385 | | 56.4 | | 297 | | 43.6 | |  |
| Boys who smoke are more or less attractive | | | | | | | | | | | | | | | | |  |
| Less attractive or no difference | 289 | | 20.8 | | 1097 | | 79.2 | | 586 | | 56.7 | | 448 | | 43.3 | |  |
| More attractive | 20 | | 51.3 | | 19 | | 48.7 | | 30 | | 61.2 | | 19 | | 38.8 | |  |
| Girls who smoke are more or less attractive | | | | | | | | | | | | | | | | |  |
| Less attractive or no difference | 303 | | 21.6 | | 1101 | | 78.4 | | 533 | | 54.1 | | 452 | | 45.9 | |  |
| More attractive | 6 | | 28.6 | | 15 | | 71.4 | | 83 | | 84.7 | | 15 | | 15.3 | |  |
